# Lipid Vesicles Loaded with an HIV-1 Fusion Inhibitor Peptide as a Potential Microbicide

**DOI:** 10.3390/pharmaceutics12060502

**Published:** 2020-05-31

**Authors:** Elena Sánchez-López, Anna Paús, Ignacio Pérez-Pomeda, Ana Calpena, Isabel Haro, María José Gómara

**Affiliations:** 1 Department of Pharmacy, Pharmaceutical Technology and Physical Chemistry, Faculty of Pharmacy, University of Barcelona, 08028 Barcelona, Spain; anacalpena@ub.edu; 2 Institute of Nanoscience and Nanotechnology (IN2UB), University of Barcelona, 08028 Barcelona, Spain; 3 Centro de Investigación Biomédica en Red de Enfermedades Neurodegenerativas (CIBERNED), University of Barcelona, 08028 Barcelona, Spain; 4 Unit of Synthesis and Biomedical Applications of Peptides, Department of Biological Chemistry, IQAC−CSIC, Jordi Girona 18, 08034 Barcelona, Spain; annapausfrutos@gmail.com (A.P.); ippnqb@cid.csic.es (I.P.-P.); isabel.haro@iqac.csic.es (I.H.); mariajose.gomara@iqac.csic.es (M.J.G.)

**Keywords:** microbicides, drug delivery system, nanoparticle, fusion inhibitor peptide, vaginal mucosa

## Abstract

The effective use of fusion inhibitor peptides against cervical and colorectal infections requires the development of sustained release formulations. In this work we comparatively study two different formulations based on polymeric nanoparticles and lipid vesicles to propose a suitable delivery nanosystem for releasing an HIV-1 fusion inhibitor peptide in vaginal mucosa. Polymeric nanoparticles of poly-d,l-lactic-co-glycolic acid (PLGA) and lipid large unilamellar vesicles loaded with the inhibitor peptide were prepared. Both formulations showed average sizes and polydispersity index values corresponding to monodisperse systems appropriate for vaginal permeation. High entrapment efficiency of the inhibitor peptide was achieved in lipid vesicles, which was probably due to the peptide’s hydrophobic nature. In addition, both nanocarriers remained stable after two weeks stored at 4 °C. While PLGA nanoparticles (NPs) did not show any delay in peptide release, lipid vesicles demonstrated favorably prolonged release of the peptide. Lipid vesicles were shown to improve the retention of the peptide on ex vivo vaginal tissue in a concentration sufficient to exert its pharmacological effect. Thus, the small size of lipid vesicles, their lipid-based composition as well as their ability to enhance peptide penetration on vaginal tissue led us to consider this formulation as a better nanosystem than polymeric nanoparticles for the sustained delivery of the HIV-1 fusion inhibitor peptide in vaginal tissues.

## 1. Introduction

HIV constitutes a disease affecting more that 40 million people worldwide [1]. Due to its severity and high prevalence, several strategies have been described for preventing this pathological infection disease. Among all, vaginal microbicide therapies to prevent sexual transmission of HIV from men to women are being currently studied [2]. In this sense, tenofovir constitutes a potential strategy to decrease the incidence of sexually transmitted HIV [3,4]. However, it does not completely prevent HIV infections [4]. Moreover, 5-Chloro-3-(phenylsulfonyl) indole-2-carboxamide has been formulated into a vaginal gel also showing promising preclinical results [5,6].

In this area, HIV-1 fusion/entry inhibitors have attracted interest as promising microbicides (compounds that can be applied inside the vagina or rectum to protect the individuals against sexually transmitted infections including HIV) since they can prevent HIV transmission by inhibiting viral entry into the host cell [7]. Particularly, fusion inhibitor peptides deserve special attention because, unlike organic molecules of low molecular weight, they can mimic the structure of the domains of the proteins, being large enough to specifically inhibit protein–protein interactions. In recent years, some preclinical studies have been carried out with fusion inhibitor peptides (C34, T20, T1249, L’644, Sifuvirtide, Albuvirtide), which target gp41 glycoprotein, for their possible use as microbicides [8,9,10]. These studies have shown that the effective use of fusion inhibitor peptides against cervical and colorectal tissue infections require the development of sustained release formulations [11]. Regarding the development of peptides able to inhibit viral entry into host cells, our research group have defined a fusion inhibitor peptide with a broad spectrum of anti-HIV-1 activity [12]. This peptide, namely E1P47, has demonstrated similar antiviral activity against HIV-1 viruses from different subtypes and different tropisms (clades A, B, C, D and AE) [12]. Despite showing antiviral activity, this short peptide is susceptible to degradation by human plasma proteases. Moreover, peptide penetration across the vaginal tissue is an important issue that needs to be overcome to achieve suitable therapeutic effects. In addition, peptide adverse effects in genital epithelial such as disruption or inflammation processes also need to be addressed without compromising the peptide pharmacological activity [13]. In this sense, it is well known that the use of nanosystems for the prolonged release of antiretroviral drugs significantly increases their efficacy and therapeutic safety and it provides stability to the active molecules that they transport, protecting them against proteolytic degradation and maintaining their prolonged release at the specific target site [13,14,15,16,17]. A valid strategy to avoid the instability of fusion inhibiting peptides in the vaginal environment, characterized by a low pH and the presence of abundant proteolytic enzymes, is the preparation of polymeric nanoparticles (NP) of poly-d,l-lactic-co-glycolic acid (PLGA). PLGA is a biodegradable polymer approved by the Food and Drug Administration (FDA) and accepted as a vehicle for drug delivery through the main routes of administration. In an attempt to overcome the well-known drawbacks of synthetic peptides for this specific therapeutic application—mainly, short half-life and rapid clearance—polymeric NPs have previously been prepared to incorporate and release the E1P47 inhibitor peptide inside vaginal mucosa [18]. Specifically, it has been shown that after topical vaginal application, these NPs are able to reach the basal layer of the cervical epithelium, which is a critical component in the process of HIV infection [11,13].

On the other hand, the use of lipid vesicles as suitable nanosystems for managing hydrophobic entry-inhibitor peptides as putative microbicides has been described. The incorporation of peptides into lipid vesicles reduces their proteolytic degradation thereby increasing their stability. Having in mind the hydrophobic nature of this inhibitor peptide, the development of lipid vesicles as peptide nanocarriers can also be considered as a potential formulation for delivering hydrophobic peptides that have a poor solubility in physiological conditions. In addition, these lipid nanosystems facilitate the targeted delivery of the fusion inhibitor peptides to the membrane subdomains where the process of entry of the HIV-1 virus into the cell takes place [19,20].

Based on the peptide E1P47 in this work we comparatively study two different formulations based on polymeric NPs and lipid vesicles in order to establish which system is the most effective for releasing the inhibitor peptide with antiviral activity in the vaginal mucosa. To this end, polymeric nanoparticles composed of PLGA and large unilamellar vesicles of palmitoyl-2-oleoyl-sn-glycero-3-phosphocholine (POPC) both loaded with the E1P47 inhibitor peptide were prepared.

Phosphatidylcholine lipids constitute more than half of the phospholipids of most eukaryotic cellular and subcellular membranes and among them, POPC is the most abundant being one of the primary constituents of the cell membranes, thus ensuring the safety of the nanocarrier [21,22]. In order to study the physical properties of the nanocarriers, the formulations were characterized in terms of size, polydispersity index, zeta potential and entrapment efficiency. Short-term stability and in vitro release of the peptide from the drug delivery systems were studied to confirm the prolonged peptide release. Finally, in vitro and ex vivo permeation assays were also carried out in order to ensure the ability of the formulations to deliver E1P47 peptide in the vaginal tissue.

## 2. Materials and Methods

### 2.1. Synthesis of E1P47 Peptide

The E1P47 peptide has been synthesized manually by solid-phase peptide synthesis as previously described [12].

### 2.2. Preparation of Large Unilamellar Vesicles

Large unilamellar vesicles (LUV) of phospholipid palmitoyl-2-oleoyl-sn-glycero-3-phosphocholine (POPC) (Avanti Polar-Lipids, Alabaster, AL, USA) containing E1P47, POPC (E1P47) LUVs, were prepared at 4 mM. POPC was dissolved in 1 mL of chloroform/methanol 2:1 (v/v). Then, the lipid mixture was dried by evaporation under vacuum. A thin film was formed and afterwards it was lyophilized overnight. The following day, it was resuspended in phosphate-buffered saline (PBS, pH 7.4) buffer with E1P47 at 0.5 mg/mL and 3% of dimethyl sulfoxide (DMSO). The mixture was sonicated obtaining a white suspension, formed by multilamellar vesicles (MLVs) which were repeatedly frozen and thawed, obtaining frozen and thawed multilamellar vesicles (FTMLV). To prepare POPC (E1P47) LUVs, FTMLV were extruded in a high-pressure extruder (Extruder, Northern Lipids Inc., Burnaby, BC, Canada) 2-times through 200 nm and 5-times through 100 nm pore-size polycarbonate filters (Nucleopore, Pleasanton, CA, USA) [23,24,25]. Empty LUVs were prepared following the same procedure but without E1P47 peptide.

### 2.3. Preparation of Polymeric Nanoparticles

PLGA nanoparticles containing E1P47 peptide, namely PLGA (E1P47) NPs, were prepared by using the solvent displacement method [26,27]. Briefly, 24 mg of PLGA (Boehringuer Ingelheim^®^) and 2 mg of E1P47 were weighed and dissolved in 2 mL of acetone. The organic solution was added dropwise into 6 mL of 10 mg/mL of polyvinyl alcohol (PVA) aqueous solution under magnetic stirring. Afterwards, acetone was evaporated under reduced pressure and PLGA (E1P47) NPs were washed 4-times by centrifugation at 14,000× *g* at 4 °C for 45 min. The final colloidal suspension was concentrated to the desired volume. Empty PLGA NPs were prepared following the same procedure but without E1P47 peptide.

### 2.4. Characterization of Formulations

#### 2.4.1. Particle Size, Zeta Potential and Polydispersity

Particle size (Z_av_) and polydispersity index (PI) were determined by dynamic light scattering using a Zetasizer nano ZS (Malvern instruments) at 25 °C and scattering angle of 90°. PI describes the width of the assumed Gaussian distribution relative to its mean. Therefore, values <0.2 are typically interpreted as monodisperse.

Zeta potential (ZP) was determined by laser-doppler electrophoresis using the same instrument. Samples were previously diluted 1:10 (v/v) in water and the assays were carried out by triplicate [28].

#### 2.4.2. Entrapment Efficiency (EE)

The amount of E1P47 entrapped in PLGA (E1P47) NPs and POPC (E1P47) LUVs was calculated indirectly determining the free E1P47 (non-encapsulated) by HPLC analytical method using a standard curve of E1P47 and applying the Equation (1) [29]. The nanosystems were diluted 1:10 (v/v) in water. Free E1P47 peptide was separated from E1P47-loaded nanosystems after centrifugation of the suspensions with centrifugal filters (Amicon Ultra 3 KDa MWCO, Millipore, Merck) at 12,000× *g* for 30 min. The amount of free E1P47 in the supernatant was determined by high-performance liquid chromatography (HPLC) analysis on a 1260 Infinity chromatograph (Agilent Technologies) using an Eclipse Plus C_18_ column (Agilent, 3.5 μm, 4.6 × 100 mm). A linear gradient (5–95%) of 0.05% trifluoroacetic acid (TFA) in acetonitrile (ACN) (solvent B) into 0.05% TFA in water (solvent A) over 20 min at a 1 mL/min flow rate was used and eluted peaks were detected at 220 nm [12]. A standard curve of E1P47 peptide covering a range from 4 µg/mL to 63 µg/mL of peptide and including a total of eight standard concentrations was analyzed. The linearity study verified that the sample solutions were in a concentration range where analyte response was proportional to the concentration. Linearity was established by calculation of a regression line from the graphical plot of the chromatographic peak area versus E1P47 standard concentration obtaining a calibration curve. Linearity was studied by calculating the regression equation and the correlation coefficient (r^2^) of the calibration curve. The calibration curve and correlation coefficient were y = 31,990.2x − 5.6 and 0.9998 respectively.

E1P47 entrapment efficiency (EE) was calculated using the equation below:

(1) $$\mathrm{EE}\;\left(\%\right)=\frac{\mathrm{Total}\;\mathrm{amount}\;\mathrm{of}\;E1P47-\mathrm{Free}\;E1P47}{\mathrm{Total}\;\mathrm{amount}\;\mathrm{of}\;\mathrm{peptide}}\times 100$$

### 2.5. Short-Term Stability of LUVs and PLGA Nanoparticles

Short-term stability of the developed drug delivery systems, PLGA (E1P47) NPs and POPC (E1P47) LUVs, was studied at 4 °C for the first 40 days of storage by recording their Z_av_ and PI.

### 2.6. Toxicity Assessment

In order to study the possible toxicity of the formulations, the MTT assay was carried out using the HEC-1A cell line [30,31]. Briefly, HEC-1A cells were seeded in 96-well plates with an initial density of 25,000 cells per well in 100 μL of culture medium (McCoy’s 5A Medium modified with 10% fetal bovine serum (FBS)) and incubated for 24 h at 37 °C and 5% CO. Cell monolayers were washed with PBS and then 100 μL of E1P47 were added in concentrations ranged from 5 μM to 0.04 μM. The plates were kept in incubation for 24 h and then the cells were incubated for 3 h with 1 mg/mL of 3-(4,5-dimethylthiazol-2-yl)-2,5-diphenyltetrazolium bromide (MTT), dissolved in culture medium without FBS. Subsequently, 100 μL of DMSO were added to each well and the plates were stirred for 30 min at room temperature. Finally, the absorbance at 560 nm was measured in a microplate reader (SpectraMax M5, Molecular Devices) and the results were expressed as a percentage relative to the absorbance of the DMSO controls.

### 2.7. Permeability Assessment

In vitro permeability assays were carried out in a dual chamber system (12-well Costar^®^ Transwell), consisting of an apical and a basal chamber separated by a monolayer of HEC-1A cells. Around 100,000 cells were seeded in membrane inserts and were cultured for 10 days [32,33]. Two buffers were used for the assay: an apical buffer, acidified Hank’s Balance Salt Solution (HBSS with 25 mM glucose, 50 mM acetate buffer, pH 4.2), and a basal buffer, neutral HBSS (HBSS with 25 mM glucose, 10 mM HEPES, pH 7.4). After washing the HEC-1A cells monolayer with PBS, the inserts were incubated for 1 h with 600 μL of basal buffer and 200 μL of apical buffer in their corresponding chambers. Later, 200 μL of E1P47 0.1 mg/mL solution were placed in apical chamber and 600 μL of basal buffer were replaced in the basal chamber. The plates were incubated for 1 h at 37 °C and 5% CO_2_ with orbital shaking at 200 rpm. After that, samples were withdrawn from both chambers and reserved for further analysis. To measure the intracellular accumulation of the compounds, HEC-1A cells were washed 2-times with PBS and E1P47 was extracted with a 70:30 methanol: water mixture. Afterwards, the samples were desalted with Pierce^®^ C_18_ Pipette Tips (Thermo Scientific, Waltham, MA, USA). E1P47 concentration from the apical, basal and intracellular compartments was determined by UPLC-MS/MS following the methodology described in Section 2.1. The results were expressed as a percentage fraction of the peptide present in the apical chamber, basal chamber and internalized in HEC-1A monolayer (intracellular).

### 2.8. Monolayer Integrity Assessment

Monolayer integrity was determined measuring the fluorescence of the Lucifer Yellow reagent (LY) [34,35]. The inserts were carefully washed twice with PBS at 37 °C and incubated for 20 min at this temperature. Subsequently, PBS was completely removed from the inserts in both apical and basal chambers. A volume of 600 μL of basal buffer (neutral HBSS) was added to the basal chamber and 200 μL of LY 500 μg/mL were added to the apical chamber. Afterwards, the plate was incubated at 37 °C with orbital shaking at 200 rpm for 60 min. Then, the inserts with cell monolayers were removed from the plates and fluorescence measurements were obtained at λ_ex_ = 428 nm and λ_em_ = 536 nm. The results were expressed as the fluorescence intensity measured in the wells versus LY control (experiment without cell monolayer with addition of LY reagent).

### 2.9. In Vitro Release Study

To study the in vitro drug release of the peptide from PLGA (E1P47) NPs and POPC (E1P47) LUVs, Franz diffusion cells were used [36]. The donor compartment and the receptor compartment were separated by a cellulose membrane with a diffusional area of 0.632 cm^2^. The receptor chamber was filled with 5 mL of transcutol^®/^H_2_O 1:1 (v/v) and kept at 37 °C under continuous stirring. A volume of 0.4 mL of each formulation was placed in the corresponding donor compartment E1P47 dissolved in transcutol^®^/H_2_O 1:1 (v/v), PLGA (E1P47) NPs and POPC (E1P47) LUVs). The samples were assessed by duplicate. The system was kept at 37 °C under constant magnetic stirring. Volumes of 0.3 mL of the receptor compartment were withdrawn at predetermined time intervals and replaced with the same volume of receptor medium. To determine the peptide remaining in the cellulose membrane, E1P47 was extracted from the membrane with ACN/H_2_O 1:1 (v/v) for 30 min under sonication. All the samples were analyzed by UPLC-MS/MS.

### 2.10. Ex Vivo Permeation Study

The ex vivo permeation study was carried out with fresh porcine vaginal mucosa from two animals provided by the Animal Facility at Bellvitge Campus University (University of Barcelona, Spain). The experiment was carried out under the protocol approved by the Animal Experimentation Ethics Committee of the University of Barcelona (Spain) and the Committee of Animal Experimentation of the regional autonomous of Catalonia (Spain).

The tissues were mounted in membrane holders with a diffusion area of 0.632 cm^2^ [36]. Franz-type diffusion cells were used, and the membrane holder was mounted between the two compartments with the epithelium side facing the donor chamber and the connective tissue region facing the receptor chamber. Transcutol^®^/H_2_O 1:1 (v/v) was used in the receptor compartment. A volume of 0.4 mL of each sample was added by duplicate at a 0.35 mg/mL, 0.39 mg/mL and 0.34 mg/mL concentration of free E1P47 (dissolved in Transcutol^®^/H2O 1:1 (v/v)), PLGA (E1P47) NPs and POPC (E1P47) LUVs, respectively to the donor compartment. Thus, the amount of peptide used for each sample was 140.0 µg for free peptide, 156.0 µg for PLGA (E1P47) NPs and 136.0 µg for POPC (E1P47) LUVs. Samples from both animals were used as replicates for each formulation. The receptor compartment was incubated at 37 °C to reproduce in vivo mucosal temperature and the receptor phase was stirred continuously. At predetermined time intervals (1, 2, 3, 4, 5 and 6 h), a volume of 0.3 mL of the acceptor medium was removed and immediately replaced by the fresh receptor solution. The withdrawn supernatant was analyzed to determine the content of E1P47 peptide by UPLC-MS/MS.

The amount of peptide retained at the vaginal mucosa was also measured upon its previous extraction by treatment of the vaginal mucosa with 1 mL (V_p_) of ACN/H_2_O MilliQ 1/1 (v/v) during 30 min in an ultrasonic bath. The solution was withdrawn from the mucosa (concentration of peptide extracted from mucosa; C_p_), the vaginal mucosa was weighted (mucosa weight; W_p_) and the supernatant was analyzed by UPLC-MS/MS.

To determine the recovery of E1P47 in the mucosa, 1.2 mL (V_1_) of a solution of known concentration of E1P47 (C_0_) in transcutol^®^/H2O 1:1 (v/v) was added to the three previously weighted tissue samples (W_t_). The samples were introduced into a bath at 37 °C for 6 h, next the supernatant (non-permeabilized concentration; C_trans_) was separated from the tissue. The three tissues were weighted (W’_t_). Then 1 mL (V_2_) of ACN/H_2_O 1:1 (v/v) was added. The vaginal mucosa samples were sonicated by ultrasound for 30 min, the supernatant was removed from the tissue (C_acn_) and analyzed by UPLC-MS/MS. Therefore, C_acn_ is the concentration of peptide in the supernatant after the extraction process in order to calculate the recovery of the peptide. It corresponds with the concentration of peptide permeabilized into the vaginal tissue after the addition of a standard amount to obtain the peptide recovery percentage.

To calculate the recovery percentage of the drug in the tissue Equation (2) was applied:

(2) $$\mathrm{Recovery}\;\left(\%\right)=\frac{\frac{C_{\mathrm{trans}}\times V_{2}}{{\;W\;\prime }_{t}}}{\frac{\left(C_{0}-C_{\mathrm{acn}}\right)\times V_{1}}{W_{t}}}\times 100$$

To calculate the real amount of peptide that was retained inside the mucosa Equation (3) was applied:

(3) $$\mathrm{Quantity}\;\mathrm{of}\;\mathrm{peptide}=\frac{C_{p}\times {\;V}_{P}}{W_{p}\times \;S}\times \frac{100}{\%\;\mathrm{Recovery}}$$

The permeation parameters and the resultant of accumulative drug versus time for permeation studies were fitted by an appropriate model using the WinNonlin computer program (Pharsight 5.2, Mountain View, CA, USA).

### 2.11. Quantification of E1P47 by UPLC-MS/MS

Chromatographic separation was performed with an Acquity UPLC pump and autosampler (from Waters, Milford, MA, USA) and MS/MS detection was carried out in the multiple reaction monitoring (MRM) mode using a TQD triple–quadrupole mass spectrometer (from Waters, Hertfordshire, UK) equipped with an electrospray (ESI) interface working in positive ion mode.

UPLC separations were performed by a ZORBAX RRHD SB-C8 column (2.1 × 150 mm, 1.8 µm; Agilent Technologies, Santa Clara, CA, USA). A gradient elution was applied using ACN (A) and water (B) with 0.1% formic acid in both solvents with a flow rate of 0.3 mL/min. The initial mobile phase composition was maintained at 5% of solvent A for 30 s and then changed linearly to 95% of solvent A (0.5–10 min). The injection volume was of 10 µL.

A previous optimization of voltage conditions of TQD detector was required in order to achieve the best sensitivity in E1P47 quantification. Cone voltage of 30 V was selected for monitoring parent ion at *m/z* 790, which shows the highest abundance of E1P47 (Figure S1A of Supplementary Material), and energy collision of 55 V was selected for monitoring product ion at *m/z* 159, that was used for quantification by external calibration. Furthermore, product ion at *m/z* 272 with energy collision of 35 V was selected for qualitative purpose in order to complete the multiple reaction monitoring (MRM) mode. The calibration curve was linear from 100 to 200 ng/mL.

## 3. Results and Discussion

### 3.1. Characterization of the Nanocarriers

PLGA (E1P47) NPs and POPC (E1P47) LUVs were characterized in terms of average size (Z_av_), Polydispersity index (PI) and zeta potential (ZP) as well as entrapment efficiency (EE).

As shown in Table 1, the Z_av_ of the POPC (E1P47) LUVs was around 100 nm whereas the PLGA (E1P47) NPs Z_av_ was around 240 nm. Polymeric nanoparticles with sizes between 200 and 500 nm have been described to be able to penetrate mucus down to the epithelial mucosa [37]. Both formulations have a suitable range of size for achieving an appropriate distribution in cervicovaginal mucosa with a minimal steric obstruction [38,39]. Moreover, the Z_av_ was not affected by the peptide loading and the PI values were lower than 0.1 indicating narrow particle size distributions corresponding to monomodal systems [40].

The ZP results showed negative values for PLGA NPs and neutral values for PLGA (E1P47) NPs (Table 1). As reported in many studies, the negative charge of PLGA NPs can be attributed to the presence of end carboxyl groups of the polymer on the nanoparticle surface [41]. Nevertheless, when PLGA NPs incorporated the E1P47 peptide, the NPs showed a neutral charge (Table 1). The peptide is a short sequence constituted by 18 amino acids (WILEYLWKVPFDFWRGVI) that shows a positive surface charge, as indicated in Table 1 [12]. Due to this positive charge, its incorporation in the NPs modifies the characteristic negative charge of the PLGA NPs. Moreover, modifications of the negative ZP of nanocarriers after peptide incorporation have also been observed by other authors [42]. Taking into account that the mucus layer itself is an anionic polyelectrolyte at a neutral pH value, the neutrality of PLGA (E1P47) NPs might minimize the PLGA electrostatic repulsion with the mucus layer and therefore contribute to the residence of the NPs loaded with E1P47 in the mucosa.

Regarding lipid vesicles composed of the zwitterionic lipid POPC, the ZP of the LUVs was negative (Table 1). As previously reported, some neutral liposomes exhibit non-zero ZP in an electric field even when they are dispersed at pH 7.4 due to the orientation of lipid head groups in the liposomal surface [43]. Accordingly, it seems that the phosphatidyl groups are located at the outer part of the polar surface of the lipid bilayer and choline groups hide behind the surface [43,44]. On the other hand, the positive charge of E1P47 led to decrease the negative ZP of POPC LUVs. This fact could be attributed to the E1P47 entrapment within the lipid bilayer. Based on the secondary structure of the peptide previously determined by NMR spectroscopy in dodecylphosphocholine (DPC) micelles and solved by using restrained molecular dynamics calculations, we assume that the peptide E1P47 is located inside the lipid bilayer, likely lying nearly parallel to the surface, with the negative-charged side chains oriented to the choline head group and the hydrophobic residues pointing to the micelle core [45].

The EE was assessed indirectly determining by analytical HPLC method the non-entrapped E1P47 using a standard curve of the peptide concentration. The E1P47 content in POPC (E1P47) LUVs was 93% from the initial drug concentration, higher than in PLGA (E1P47) NPs (69%). Therefore, in PLGA NPs 300 μg/mL of E1P47 peptide are encapsulated whereas in LUVs 465 μg/mL are loaded. The results obtained by PLGA NPs are in accordance with other authors [14]. In this sense, the solvent displacement method seems to be an efficient production method able to encapsulate a high amount of peptide. The higher entrapment efficiency of E1P47 in POPC vesicles is probably due to the hydrophobic nature of the inhibitor peptide. Thus, the peptide prefers to remain soaked into the lipid bilayer of POPC vesicles than entrapped into the aqueous matrix of polymeric NPs. Moreover, our results suggest that high pressure extrusion process is an effective method to obtain LUVs of a narrow polydispersity able to load E1P47 peptide.

### 3.2. Short-Term Stability of the Nanocarriers

**Figure 1:**
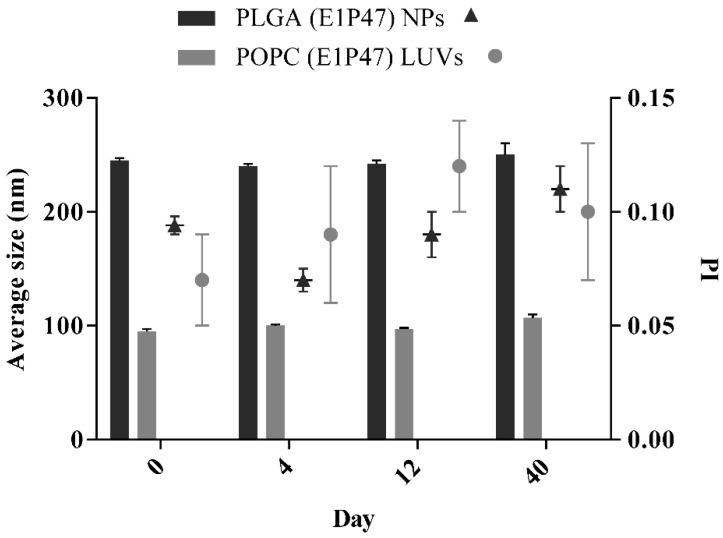
Short-term stability of PLGA (E1P47) NPs and POPC (E1P47) LUVs. Bars correspond to average size measurements and are referred to the left side of the Y axis. Symbols correspond to polydispersity index (PI) results and are referred to the right side of the Y axis. Values are expressed as mean ± SD. (Z_av_ and PI stored at 4 °C; *n* = 3).

Short-term stability was studied at 4 °C for the first 40 days of storage. PLGA (E1P47) NPs (Figure 1, Table S1 of Supplementary Material) remain stable for more than one month after their storage. However, a slight increase of particle size and PI values can be observed after 40 days. These results are in accordance with the ones reported by Wan et al. that develop PLGA NPs that were stable for at least two months after their preparation [46,47].

POPC (E1P47) LUVs were stable for the first two weeks and afterwards a significant increase in average size was recorded (*p* < 0.05). Although other authors were able to obtain an increased LUVs stability over two months, this may be due to the fact that their PI values are the double of the PI obtained in the present work [48].

The limited stability of nanocarriers in aqueous suspension is well known and these results confirm that in order to improve long-term stability, the removal of water from the solution (either by freeze-drying or by spray-drying) would be necessary [27,49].

### 3.3. In Vitro Drug Release

To evaluate the efficacy of E1P47 delivery of PLGA (E1P47) NPs and POPC (E1P47) LUVs, release studies were comparatively carried out. E1P47 release from the nanocarriers was studied at 37 °C using Franz diffusion cells. In vitro release profiles of the preparations were fitted to a mono-compartmental model [50,51]. This profile corresponds to a biopharmaceutical model, where peptide entry and elimination occur simultaneously. Previous to the in vitro drug release experiments, peptide degradation was confirmed. In this sense, the peptide was incubated in the in vitro drug release media and 17% of the peptide was degraded during the first 8 h of incubation (Figure S2 of Supplementary Material). This result was taken into account when the biopharmaceutical data derived from the in vitro release assay was analyzed. Therefore, the mono-compartmental model was applied and the parameters studied were the entry rate constant (K_a_), the elimination rate constant (K_10_), the maximal drug level contained (Q_max_), the time at which Q_max_ occurs (T_max_) and the time from drug administration to its appearance on medium (L_time_).

**Figure 2:**
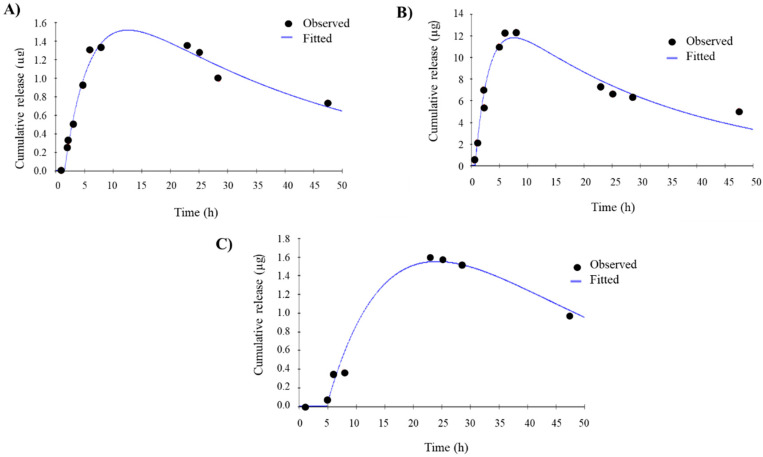
In vitro release profile, measured mass of released peptide (black circles) fitted to a mono-compartmental model (blue lines). (**A**) Mono-compartmental fitted model of free E1P47; (**B**) Mono-compartmental fitted model of PLGA (E1P47) NPs; (**C**) Mono-compartmental fitted model of POPC (E1P47) LUVs.

The observed release profile and the pharmacokinetic parameters of the free E1P47 are shown in Figure 2A and Table 2, respectively. The entry rate constant and the elimination rate constant were (1.9 ± 0.6) × 10^−1^ h^−1^ (K_a_) and (6.0 ± 4.9) × 10^−2^ h^−1^ (K_10_), respectively. The entry of peptide started at 1.4 ± 0.1 h (L_time_) and reached maximum concentration of E1P47 at 11.1 ± 2.1 h (T_max_).

Figure 2B shows the release profiles of E1P47 from PLGA (E1P47) NPs and the biopharmaceutical parameters are detailed in Table 2. As it can be observed, the entry rate constant (K_a_) was higher than the free peptide and the elimination rate constant was considerably lower (2.8 ± 0.4) × 10^−2^ h^−1^ (K_10_). These results may indicate a protection exerted by PLGA NPs avoiding peptide degradation processes. The maximal drug found in the receptor media was observed at 7.0 ± 3.4 h (T_max_), subsequently decreasing the amount of released peptide. This T_max_ time was shorter than those of the POPC (E1P47) LUVs, thus indicating that the peptide release profile is longer lasting when using the liposomes as nanocarriers. The release profile obtained for the nanoparticle formulation was very similar to that observed for the free peptide, showing a rather increased release, higher in the case of the PLGA (E1P47) NPs. E1P47 release depended on the nature of the delivery system. In our case, the peptide was uniformly distributed or dissolved in the matrix of PLGA (E1P47) NPs and its release took place by diffusion or erosion of the matrix. Rapid initial release was due to burst effect, attributed to the fraction of the drug which was adsorbed or weakly bound to the large surface area of the PLGA (E1P47) NPs [52,53].

The release profile of E1P47 from POPC (E1P47) LUVs (Figure 2C) was sustained over time with a rate entry constant of (1.1 ± 0.5) × 10^−1^ h^−1^. The maximum E1P47 release level was at 23.0 ± 1.7 h and the lag time was at 3.0 ± 2.5 h. These results describe a slow release of the peptide due to the hydrophobic nature of the peptide favoring its entrapment into the lipid bilayer [54].

The amount of peptide used for each sample was 140 µg for free peptide, 156 µg for PLGA (E1P47) NPs and 136 µg for POPC (E1P47) LUVs. The maximal amount of peptide found in the medium (Q_max_) was 1.8 ± 0.4 µg, 8.0 ± 5.4 µg and 1.5 ± 0.1 µg (Table 2) respectively for each formulation. This represents a very small amount of peptide found in the receptor phase probably due to degradation phenomena. Thus, a peptide extraction of each membrane was carried out to quantify the peptide that might have been retained in them (Table 3). While the free peptide was only retained in a low percentage in the membrane (0.1%), PLGA (E1P47) NPs were able to increase its retention on the membrane in a higher amount (1.5%). This value is greater than the retention of the peptide released by the POPC (E1P47) LUVs (1.0%). As expected, free peptide is not retained by the membrane whereas PLGA and POPC show to retain it. In this sense, this might be due to the fact that the NPs containing the peptide are attached to the membrane and for this a high amount of peptide is found. In this sense, the affinity of PLGA synthetic polymer to the dialysis membrane seems to be higher than the POPC LUVs. Moreover, LUVs reduced size compared with PLGA NPs may also contribute to their lower retention in the membrane pore.

Therefore, increased in vitro retention is provided by both formulations. In spite of these, results do not take into account the mucus barrier of vaginal tissue which is the main obstacle for vaginal penetration, we can observe a fast release of the peptide from PLGA (E1P47) NPs and a higher retention in the dialysis membrane whereas POPC (E1P47) LUVs show a slow peptide release with lower retention on the membrane.

### 3.4. In Vitro Permeability Assay

**Figure 3:**
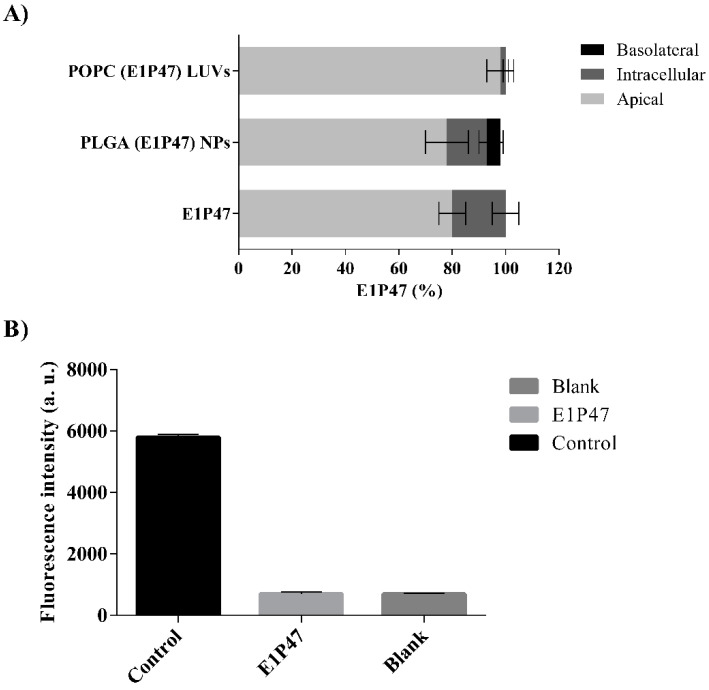
In vitro permeability assay in HEC-1A cells. (**A**) Percentage of E1P47 peptide quantified in each compartment of the Transwell system after treatment with POPC (E1P47) LUVs, PLGA (E1P47) NPs and E1P47, respectively; (**B**) Lucifer Yellow leakage through HEC-1A monolayer after treatment with E1P47 peptide and without the peptide (blank). The experiment was also carried without cell monolayer (control) to obtain the total leakage of Lucifer Yellow thorough the Transwell membrane inserts.

In order to investigate the possible application of E1P47 peptide as a microbicide, a permeability assay to test the potential of vaginal permeation of E1P47 peptide in a cell line originated from a human endometrial carcinoma (HEC-1A) was used [32,33]. First, the viability of HEC-1A cells was evaluated after treatment with E1P47 peptide. A linear range of concentrations (0.04–5 µM) was assayed. This range was selected taken into account the results of the HIV-1 antiretroviral cellular assays previously reported (IC_50_ = 3 µM) [12]. The viability percentages were higher than 90% in all the concentrations tested demonstrating that the peptide was not toxic in the range of the studied concentrations [12]. The permeability assessment demonstrated that most of E1P47 was located in the apical zone and a low percentage remained inside the cellular monolayer. Free peptide was not found in the basolateral zone, indicating that it is not able to cross the cellular monolayer (Figure 3A). Moreover, once the permeability assay was performed, a monolayer integrity assessment was required to ensure the stability of HEC-1A monolayer and to confirm that no damage or ruptures of the monolayer occurred. For this purpose, Lucifer Yellow (LY), a paracellular marker, easily detectable by fluorescence was used to check the tight junctions of the cell monolayer [55]. LY demonstrated very low permeability. Specifically, results obtained in two independent assays showed that integrity percentage was close to 80% which could be considered acceptable after experimental manipulation of inserts with cell monolayers. These results indicate that E1P47 did not disrupt the cellular monolayer [34].

The PLGA (E1P47) NPs and the POPC (E1P47) LUVs were also evaluated. PLGA (E1P47) NPs increased the peptide transport across the monolayer much more efficiently than lipid vesicles.

In addition, the free peptide and the PLGA (E1P47) NPs showed the ability to remain in the cell monolayer. These results can be attributed both to the hydrophobic characteristics of the E1P47 peptide as well as to the properties of the polymer. On the other hand, after applying POPC (E1P47) LUVs to the cell monolayer, almost all of the peptide was found in the apical compartment (Figure 3A). According to the results of the in vitro release parameters of the POPC (E1P47) LUVs, the scarce amount of E1P47 peptide found in the cell monolayer could be attributed to the sustained release of the E1P47 peptide from the liposomal carrier since after one hour of exposure the peptide has not been released from liposomes.

### 3.5. Ex Vivo Permeation

In order to study the permeation of the PLGA (E1P47) NPs and POPC (E1P47) LUVs in the vaginal tissue, an ex vivo permeation assay was carried out using porcine vaginal mucosa. Generally, porcine vaginal mucosa is considered a suitable permeability model for human vaginal mucosa, especially due to their similar lipid composition [56]. After 6 h of experiment (maximum time of tissue viability), no peptide E1P47 was found on the receptor media upon the application of neither the free peptide nor any of the nanocarrier formulations loaded with E1P47. It should be noted that some drugs can permeate the vaginal mucosa in concentration enough to possess systemic effects, which would be undesirable for the E1P47 local application purpose [57]. Therefore, since no peptide was found in the receptor media, probably due to peptide degradation processes, this may be an indicator of low bloodstream peptide permeation suitable for local therapeutic effects.

Hence, to determine the amount of the peptide retained in the mucosa, several steps were followed. Firstly, peptide recovery was calculated applying the Equation (2), described in ex vivo permeation study from the experimental section. Therefore, a peptide recovery of 24 ± 3% was obtained. This percentage is lower than those obtained by other authors studying drug recovery from vaginal tissue, but it allowed us to achieve a suitable selectivity avoiding interferences caused by proteins, tissue compounds and other peptides [58].

In the ex vivo assay an initial concentration (C_o_) of 41 μg/mL of each formulation was added to each mucosa. The quantity of peptide retained in the mucosa was calculated applying Equation (3). The concentration of peptide extracted from the mucosa (C_p_), weight of the mucosa (W_p_) and the actual quantity of peptide are shown in Table 4. The quantity of peptide retained in the vaginal tissue after adding PLGA (E1P47) NPs (3.6 (µg/g) cm^−^^2^) was lower than upon adding the peptide without any nanocarrier (10 (µg/g) cm^−2^) suggesting that the PLGA (E1P47) NPs were not able to enter the vaginal tissue. Considering the hydrophobic nature of the peptide loaded PLGA nanoparticles, in the present form they would not represent a suitable formulation for vaginal delivery since the vaginal mucus shows high affinity with positively charged particles [59,60]. Therefore, in order to achieve suitable vaginal retention values using PLGA NPs, additional surface modifications, permeation enhancers or mucoadhesive excipients would be necessary [60,61]. However, POPC (E1P47) LUVs demonstrated the ability to enhance the E1P47 peptide retention (40.0 µg/g × cm^−2^) showing a 4-fold higher retention than the obtained upon the application of E1P47 without nanocarrier. In this sense, POPC (E1P47) LUVs would probably exert a mucoadhesive effect not present in the PLGA (E1P47) NPs. These NPs may be trapped by the lumenal mucus layer either through adhesive or steric interactions being unable to penetrate into vaginal tissue [60,61]. On the other hand, due to the LUVs reduced size and POPC presence in biological membranes, they demonstrated the ability to enhance E1P47 penetration into vaginal tissue [62]. These results indicate the localization of the nanocarriers at the upper-layers of the epithelial mucosa since almost no peptide was able to permeate to the receptor chamber but it was internalized in the vaginal tissue. Of note, the peptide may be released at the site of HIV transmission [13].

As previously reported, the peptide concentration required for inhibiting the replication of HIV-1 in cell cultures is in the range of low micromolar (IC_50_ = 3 µM and IC_90_ below 10 µM for HIV-1_NL4-3_) [12]. The peptide concentration retained in the vaginal tissue upon adding POPC (E1P47) LUVs was 11.0 ± 2.0 µM (Table 4) which was higher than the IC_50_ and IC_90_ required for its antiviral activity. Therefore, POPC (E1P47) LUVs would be able to deliver peptide concentrations in the vaginal tissue that should be sufficient to exert the pharmacological effect. Thus, the small size of POPC LUVs and their lipid-based composition as well as their ability to enhance E1P47 penetration on vaginal tissue led us to consider POPC (E1P47) LUVs as a better nanosystem than PLGA (E1P47) NPs for the delivery of this HIV-1 fusion inhibitor peptide in vaginal tissues. Moreover, it has been reported that the addition of cholesterol increases vesicles stability in vivo upon contact with blood, prevents lipid exchange and has an additional stabilizing effect [63,64,65]. In our hands, POPC LUVs were able to release the drug slowly achieving sufficient peptide concentration in vaginal tissue. Although the incorporation of cholesterol might be beneficial for a long-term stability of LUVs it could delay the peptide release affecting the concentrations retained in the tissue and probably causing an excessively prolonged drug release [65].

The development of biodegradable and biocompatible nanocarrier-based formulations for local application of the antiviral peptide might contribute to advancement in HIV therapy, where recent efforts have focused on disease prevention [66,67].

## 4. Conclusions

In the present study, a suitable delivery nanosystem for releasing an HIV-1 fusion inhibitor peptide in vaginal mucosa has been proposed. Polymeric biodegradable PLGA nanoparticles and lipid vesicles loaded with the inhibitor peptide showed average sizes and polydispersity index values corresponding to monodisperse systems which were appropriate for vaginal permeation. Although high encapsulation efficiency was observed in both nanosystems, POPC LUVs were able to entrap almost the entire amount of peptide added into the formulation (entrapment efficiency higher than 90%) whereas the EE of PLGA NPs was slightly lower. In vitro drug release demonstrates that both nanocarriers were able to retain E1P47 peptide on the dialysis membrane, being more effective PLGA NPs. However, POPC (E1P47) LUVs demonstrated a sustained release of the inhibitor peptide as well as the ability to enhance peptide retention on ex vivo vaginal tissues. PLGA NPs demonstrated a drug release in vitro similar to the free peptide and did not achieve significant peptide concentrations into the vaginal tissue. Importantly, none of the nanocarriers were able to permeate across the vaginal tissue thus probably avoiding adverse systemic effects in vivo. Hence, POPC (E1P47) LUVs are able to deliver a sustained inhibitor peptide concentration in the vaginal tissue high enough to exert its anti-viral function. In this work, a proof of concept has been established regarding the use of PLGA NPs and POPC LUVs loaded with anti-HIV-1 peptides for local vaginal therapy. Therefore, this study demonstrates that POPC LUVs loaded with the inhibitor peptide E1P47 might be considered as a suitable formulation for its possible application as a microbicide against HIV infection.

## Figures and Tables

**Table 1:** Average size (Z_av_), PI, ZP and EE values of PLGA (E1P47) nanoparticles (NPs) and POPC (E1P47) large unilamellar vesicles (LUVs) (*n* = 3).

Formulation	Z_av_ ± SD (nm)	PI ± SD	ZP ± SD (mV)	EE (%)
Free E1P47	-	-	10.6 ± 0.6	-
PLGA NPs	245 ± 2	0.09 ± 0.00	−13.0 ± 1.1	-
PLGA (E1P47) NPs	240 ± 2	0.07 ± 0.05	−0.5 ± 0.3	69%
POPC LUVs	98 ± 1	0.05 ± 0.02	−13.0 ± 1.0	
POPC (E1P47) LUVs	95 ± 2	0.07 ± 0.02	−3.5 ± 0.1	93%

**Table 2:** Biopharmaceutical parameters of E1P47, PLGA (E1P47) NPs and POPC (E1P47) LUVs release. Values express the mean and standard deviation of two independent assays.

Formulation	K_a_ (h^−1^)	K_10_ (h^−1^)	L_time_ (h)	Q_max_ (µg)	T_max_ (h)
Free E1P47	(1.9 ± 0.6) × 10^−1^	(6.0 ± 4.9) × 10^−2^	1.4 ± 0.1	1.8 ± 0.4	11.1 ± 2.1
PLGA (E1P47) NPs	(6.0 ± 0.8) × 10^−1^	(2.8 ± 0.4) × 10^−2^	1.8 ± 1.5	8.0 ± 5.4	7.0 ± 3.4
POPC (E1P47) LUVs	(1.1 ± 0.5) ×·10^−1^	(1.4 ± 0.6) × 10^−2^	3.0 ± 2.5	1.5 ± 0.1	23.0 ± 1.7

**Table 3:** Quantification of the peptide retained in the membrane used in the in vitro drug release assays.

Formulation	Peptide Retained in the Membrane (µg)	Peptide Retained (%)
Free E1P47	0.1 ± 0.5	0.1
PLGA (E1P47) NPs	2.2 ± 1.5	1.5
POPC (E1P47) LUVs	1.5 ± 0.5	1.0

**Table 4:** Values of the concentration of peptide extracted from the mucosa (C_p_), weight of the mucosa (WP) and quantity of peptide retained in the ex vivo permeation study. Values express the mean and standard deviation of two independent assays.

Formulation	C_p_ (µg/mL)	W_p_ (g)	Peptide Retained (Mean ± SD; µg/g × cm^−2^)	Concentration of Peptide Retained (µM)
Free E1P47	0.5 ± 0.4	0.4 ± 0.1	10.0 ± 7.0	3.0 ± 2.0
PLGA (E1P47) NPs	0.2 ± 0.1	0.4 ± 0.1	3.6 ± 0.1	1.0 ± 0.0
POPC (E1P47) LUVs	2.5 ± 0.2	0.4 ± 0.1	40.0 ± 8.0	11.0 ± 2.0

## Supplementary Materials

The following are available online at https://www.mdpi.com/1999-4923/12/6/502/s1, Figure S1: average mass spectra from E1P47 under ESI at 50 V of cone voltage, Figure S2: degradation of E1P47 at 37 ºC in Transcutol^®^/H2O 1:1 (v/v) for 48 h, Figure S3: duplicate of the in vitro release profile, measured mass of released peptide (black circles) fitted to a mono-compartmental model (blue lines), 2A) Mono-compartmental fitted model of free E1P47; 2B) Mono-compartmental fitted model of PLGA (E1P47) NPs; 2C) Mono-compartmental fitted model of POPC (E1P47) LUVs.drug release, Table S1: Short-term stability of PLGA (E1P47) NPs and POPC (E1P47) LUVs (Zav and PI values).
