# Supplementary material for: Lipid Vesicles Loaded with an HIV-1 Fusion Inhibitor Peptide as a Potential Microbicide
Source: Pharmaceutics. 2020 May 31;12(6):502. doi: 10.3390/pharmaceutics12060502 (PMC7355883; doi:10.3390/pharmaceutics12060502)
Supplement: Supplementary file 1 [file pharmaceutics-12-00502-s001.pdf]

pharmaceutics

MDPI

Supplementary Materials: Lipid Vesicles Loaded with an HIV-1 Fusion Inhibitor Peptide as a Potential Microbicide

Elena Sánchez-López *, Anna Paús, Ignacio Pérez-Pomeda, Ana Calpena, Isabel Haro and María José Gómara

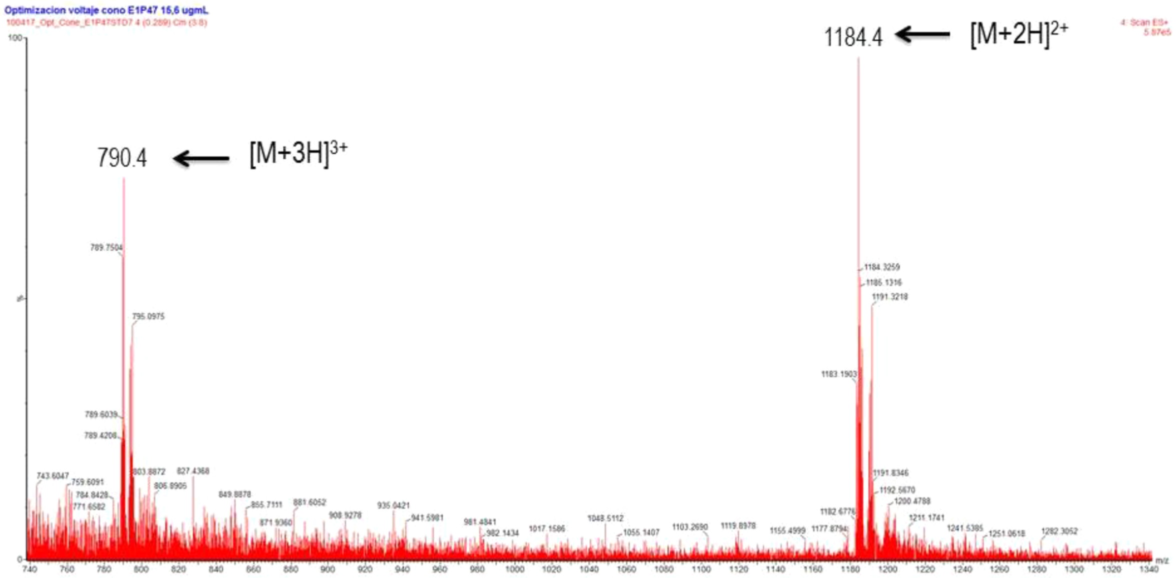

Figure S1. Average mass spectra from E1P47 under ESI at 50 V of cone voltage.

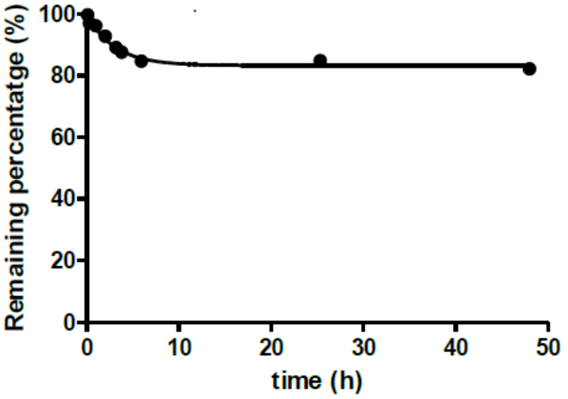

Figure S2. Degradation of E1P47 at 37ºC in Transcutol®/H2O 1:1 (v/v) for 48 hours.

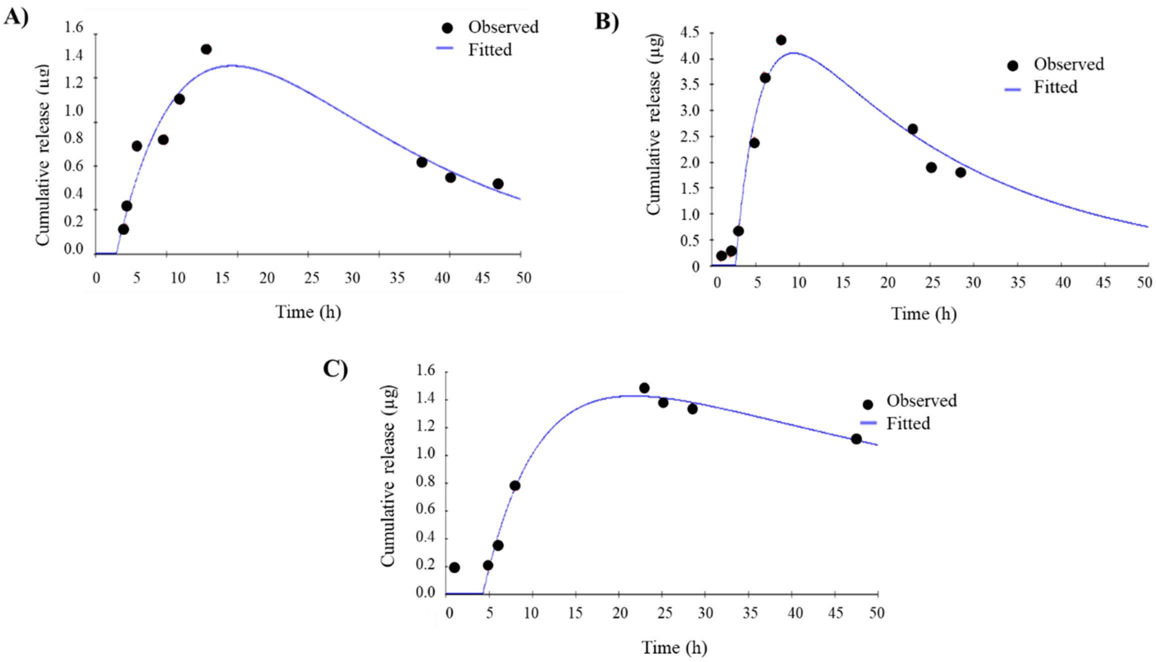

Figure S3. Duplicate of the in vitro release profile, measured mass of released peptide (black circles) fitted to a mono-compartmental model (blue lines). 2A) Mono-compartmental fitted model of free E1P47; 2B) Mono-compartmental fitted model of PLGA (E1P47) NPs; 2C) Mono-compartmental fitted model of POPC (E1P47) LUVs.drug release.

Table S1. Short-term stability of PLGA (E1P47) NPs and POPC (E1P47) LUVs (Z_{av} and PI values).

Day	PLGA (E1P47) NPs		POPC (E1P47)LUVs	
	Z_{av} ± SD (nm)	PI ± SD	Z_{av} ± SD (nm)	PI ± SD
0	245.1 ± 2.0	0.30 ± 0.00	95.1 ± 2.0	0.07 ± 0.02
4	240. ± 2.2	0.07 ± 0.05	100.1 ± 1.1	0.09 ± 0.03
12	242.4 ± 3.1	0.09 ± 0.01	97.1 ± 1.2	0.12 ± 0.02
40	250.0 ± 10.2	0.11 ± 0.01	107.4 ± 3.0	0.10 ± 0.04
